# Claudin proteins as emerging therapeutic targets for solid tumors

**DOI:** 10.1038/s41568-026-00913-3

**Published:** 2026-03-02

**Authors:** Antonio Saviano, Alberto Toso, Samuel J. Klempner, Thomas F. Baumert

**Affiliations:** 1 University of Strasbourg, Inserm, UMR_S1110, Institute of Translational Medicine and Liver Disease (ITM), 67000 Strasbourg, France; 2 Gastroenterology and Hepatology Service, Strasbourg University Hospitals, Strasbourg, France; 3 IHU Strasbourg, Strasbourg, France; 4 Alentis Therapeutics, Allschwil, Switzerland; 5 Dept. of Medicine, Division of Hematology-Oncology, Massachusetts General Hospital, Boston, MA, USA; 6 Institut Universitaire de France, Paris, France

## Abstract

Claudins (CLDNs) are transmembrane proteins that contribute to the epithelial cell polarity and integrity of tight junctions in healthy tissues. CLDNs are frequently overexpressed across different solid tumors, and expression correlates with tumor subtype, grade, and prognosis. Dysregulated CLDN expression modulates oncogenic signaling, and contributes to tumor proliferation, epithelial–mesenchymal transition (EMT), stemness, fibrosis, immune modulation and therapeutic resistance. Due to their frequent overexpression and functional role in cancer biology, CLDNs have emerged as attractive therapeutic targets. Their surface expression can be exploited to guide therapies into tumors. For example, a monoclonal antibody (mAb) targeting the CLDN18.2 isoform has reached clinical approval, validating the potential of CLDN-directed approaches. Additional strategies such as antibody–drug conjugates, bispecific and trispecific antibodies, and chimeric antigen receptor (CAR)-T cells are in development for several CLDN family members. Targeting intracellular CLDN domains or their downstream signaling to disrupt their biological function may offer further promise. Here we review the functional role of CLDN biology in solid tumors, summarize the clinical development of therapeutic approaches and discuss opportunities for biomarker-enriched patient selection. Collectively, we highlight CLDN targeting as a precision oncology approach relevant to multiple solid tumors.

## Introduction

Tight junctions are fundamental structures mediating cell-to-cell adhesion, cell polarity and signaling among epithelial cells. These multiprotein complexes consist of transmembrane components and associated cytoplasmic scaffolding and regulatory proteins. Tight junctions serve three principal functions: first, the barrier function, which seals the paracellular space and regulates ion and solute flux^1^; second, the fence function, which preserves cell polarity by preventing mixing of apical and basolateral membrane domains^2,3^ and third, the signaling function, which transmits environmental signals to adapt cell response and functions^4–6^.

**Figure 1:**
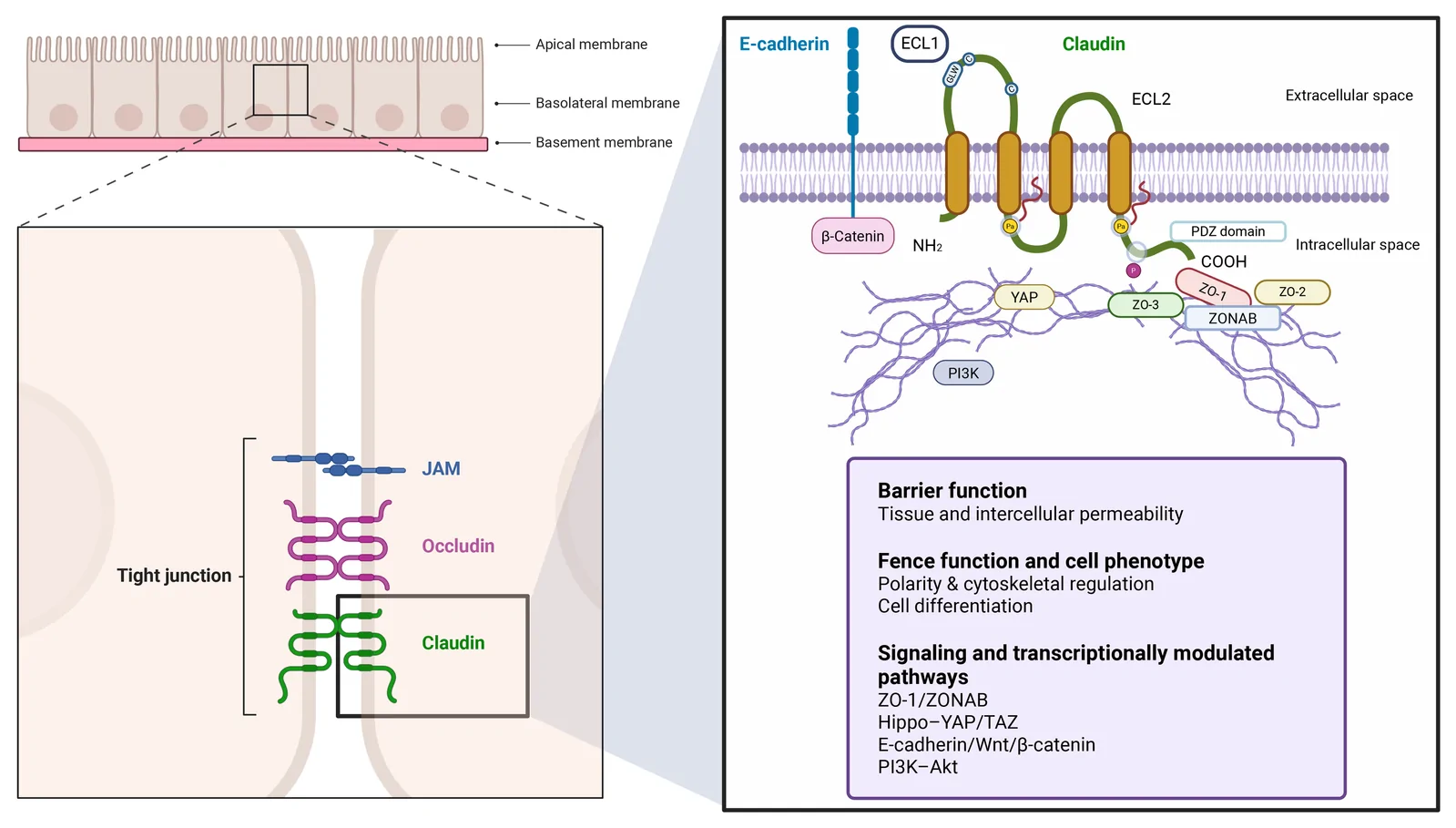
Structure and functional domains of human claudin proteins in tight junctions. Tight junctions are composed of transmembrane proteins such as claudins (CLDNs), MARVEL domain proteins such as occludin and tricellulin, blood vessel epicardial substance (BVES) and junctional adhesion molecules (JAMs). CLDNs are four-pass transmembrane proteins that form the backbone of epithelial tight junctions. Each protein comprises four α-helical transmembrane domains, two extracellular loops (ECL1 and ECL2), and cytoplasmic N-terminal and C-terminal tails. ECL1 harbors a conserved glycine, leucine, tryptophan (GLW) motif and two cysteine residues forming an intramolecular disulfide bridge, both critical for structural stability and tight junction assembly^9^. Due to its conserved structure, sequence motifs and functional relevance, ECL1 is frequently targeted in drug development. ECL2 facilitates CLDN–CLDN interactions between adjacent cells, supporting tight junction strandformation. Functionally relevant post-translational modifications include phosphorylation (P) of intracellular residues, which modulates CLDN localization, trafficking, and interaction with scaffold proteins and palmitoylation of juxtamembrane cysteines, which enhances membrane anchoring and tight junction incorporation. Most CLDNs possess a C-terminal PDZ-binding motif that mediates interactions with intracellular scaffold proteins such as zona occludens protein 1 (ZO1), ZO2, and ZO3. These complexes anchor CLDNs to the actin cytoskeleton and serve as platforms for signal integration. CLDNs contribute to paracellular permeability by forming ion-selective pores and sealing strands within tight junctions that control the passage of water and solutes between cells, and regulate epithelial polarity by acting as a “fence” that restricts the diffusion of membrane proteins and lipids between apical and basolateral domains. Moreover, CLDNs act as signal transducers through interactions with scaffolding proteins and signaling pathways. Through these interactions, they modulate key oncogenic and developmental pathways, including ZO1–ZONAB (also known as CSDA or YBX3), Hippo–Yes-associated protein (YAP) and transcriptional co-activator with PDZ-binding motif (TAZ), PI3K–AKT, and WNT–β-catenin signaling. Beyond tight junctions, CLDNs can engage with and modulate the function of other membrane proteins, such as E-cadherin and epithelial cell adhesion molecule (EpCAM), influencing cell adhesion, polarity, and fate determination. As an example, E-cadherin, together with β-catenin and α-catenin, forms the adherens junction complex that links to the actin cytoskeleton and provides a structural and signaling platform for tight junction assembly and epithelial polarity, thereby coupling CLDN-mediated barrier function with cytoskeletal dynamics and WNT signaling.

Claudins (CLDNs) are key components of tight junction-forming protein complexes and serve as structural and signaling components^7^. The name CLDN is derived from the Latin claudere, meaning “to close”^8^. In humans, the CLDN family comprises 23 members, all of which share a conserved membrane structure of four transmembrane domains^9^ (Fig. 1), but exhibit diverse tissue-specific expression patterns and distinct functional roles (Table 1).

CLDNs can be broadly categorized into barrier-forming CLDNs, which reinforce tight-junction integrity (Table 1), and channel-forming CLDNs, which generate selective paracellular ion pores^10,11^. Recent single-CLDN reconstitution studies refined this dichotomy by proposing four mechanistic classes defined by their intrinsic ability to assemble tight junction strands **[G]** and regulate ion conductance^12^. Class 1 CLDNs (e.g. CLDN1, 3, 4, 5, 6, 7, 11, 18.1, 19) act as autonomous barrier-forming proteins that independently generate tight, low-conductance junctions. Class 2 CLDNs (such as CLDN2, 10A, 10B and 15) are autonomous channel-forming proteins that assemble junctional strands but introduce ion-selective paracellular conductance. In contrast, Class 3 CLDNs (including CLDN8, 12, 16, 17 and others) are non-autonomous, as they do not form strands on their own and instead require co-expressed CLDNs to integrate into existing junctions. Class 4, represented uniquely by CLDN18.2, forms weak strands under neutral conditions but exhibits acid-enhanced junctional assembly, reflecting its physiological specialization in the stomach. This refined classification provides a mechanistic continuum that aligns classical barrier or channel functions with the intrinsic assembly properties of individual CLDNs^12^.

Beyond their structural roles, CLDNs are involved in intracellular signaling, influencing pathways related to epithelial proliferation, differentiation, and maintenance of polarity. This signaling role is primarily mediated through the PDZ-binding motif present on the cytoplasmic C-terminus of most CLDNs^13^ (Fig. 1). Under normal conditions, this motif enables interactions with intracellular scaffold proteins such as zonula occludens protein 1 (ZO1), ZO2, and ZO3, which are essential for proper CLDN localization, stabilization within the membrane, and anchorage to the actin cytoskeleton^14^. A notable example is the ZO1-associated nucleic acid-binding protein (ZONAB; also known as CSDA or YBX3) signaling axis, where CLDN-associated ZO1 binds and sequesters the transcription factor ZONAB at the membrane. Loss of tight junction integrity leads to ZONAB nuclear translocation and activation of genes involved in proliferation and inhibition of cell differentiation^15^.

In cancer, CLDNs are frequently dysregulated, with aberrant overexpression and/or redistribution on the cancer cell surface to non-junctional membrane regions making them accessible targets for therapeutic intervention. Two key points should be emphasized: first, although CLDNs have been shown to regulate a variety of cancer-related processes and act as modulators or facilitators that support tumor growth and adaptation, there is no universal pan-cancer mechanism underlying their involvement in tumor pathophysiology. Current evidence rather indicate a context- and tumor dependent mechanism of action, underscoring the need for careful analysis when considering CLDNs as therapeutic targets. Second, it is important to distinguish ´drivers´ from ´markers´. While some CLDN alterations play a functional role in cancer biology (for example CLDN1 or CLDN6 ref^16,17^) other CLDNs show simple associations or rather act as passive cell surface antigens (for example, CLDN18.2, which is used for targeted drug delivery into tumors). Although some members, such as CLDN18.2, appear to serve primarily as lineage-restricted markers, they may nonetheless exert some functional effect when overexpressed. The potential of targeting CLDNs for cancer therapy has recently been demonstrated with the approval of zolbetuximab, a mAb targeting the CLDN18.2 isoform, for the treatment of CLDN18.2-positive gastric and gastroesophageal junction cancers^18–20^.

In this Review we provide an overview of the altered expression and functional roles of CLDNs in tumor biology, summarize current progress in the clinical development of CLDN-directed therapies for solid tumors and discuss existing knowledge gaps as well as future challenges and opportunities in the development of CLDN-targeted therapies for cancer.

## Altered regulation of CLDN proteins in cancer

Claudin dysregulation in cancer primarily reflects alterations in protein expression that arise from disrupted regulatory control. These changes are driven by mechanisms acting at multiple levels, including genetic mutations, transcriptional rewiring and epigenetic modifications, which together modulate CLDN abundance, localization and downstream effects on cancer epithelial cell and tumor phenotype.

### Human CLDN expression patterns across solid tumors

A large body of evidence derived from patient tumor samples has shown that CLDN overexpression is frequent in many epithelial cancers ^17,18,21–37^. From a therapeutic perspective, the most clinically relevant CLDN expression patterns involve either marked upregulation or aberrant subcellular localization. In particular, redistribution to membrane compartments outside canonical tight junctions makes CLDNs accessible to therapeutic antibodies, enabling effective targeting^16,33^. In addition, protein isoform expression is of clinical relevance. An example is CLDN18, which is expressed as two isoforms: CLDN18.1 is predominantly expressed in the alveolar epithelium and lung tumors, whereas CLDN18.2 is confined to the gastric mucosa and is overexpressed in gastric, pancreatic, esophageal, and certain ovarian cancer and colorectal cancers (CRCs)^23,38^. These isoforms differ by 21 amino acids within the first 69 residues, including 8 substitutions in the extracellular loop 1, the primary binding site for most therapeutic antibodies, which in turn influences antibody binding and selectivity ^18,39^.

**Figure 2:**
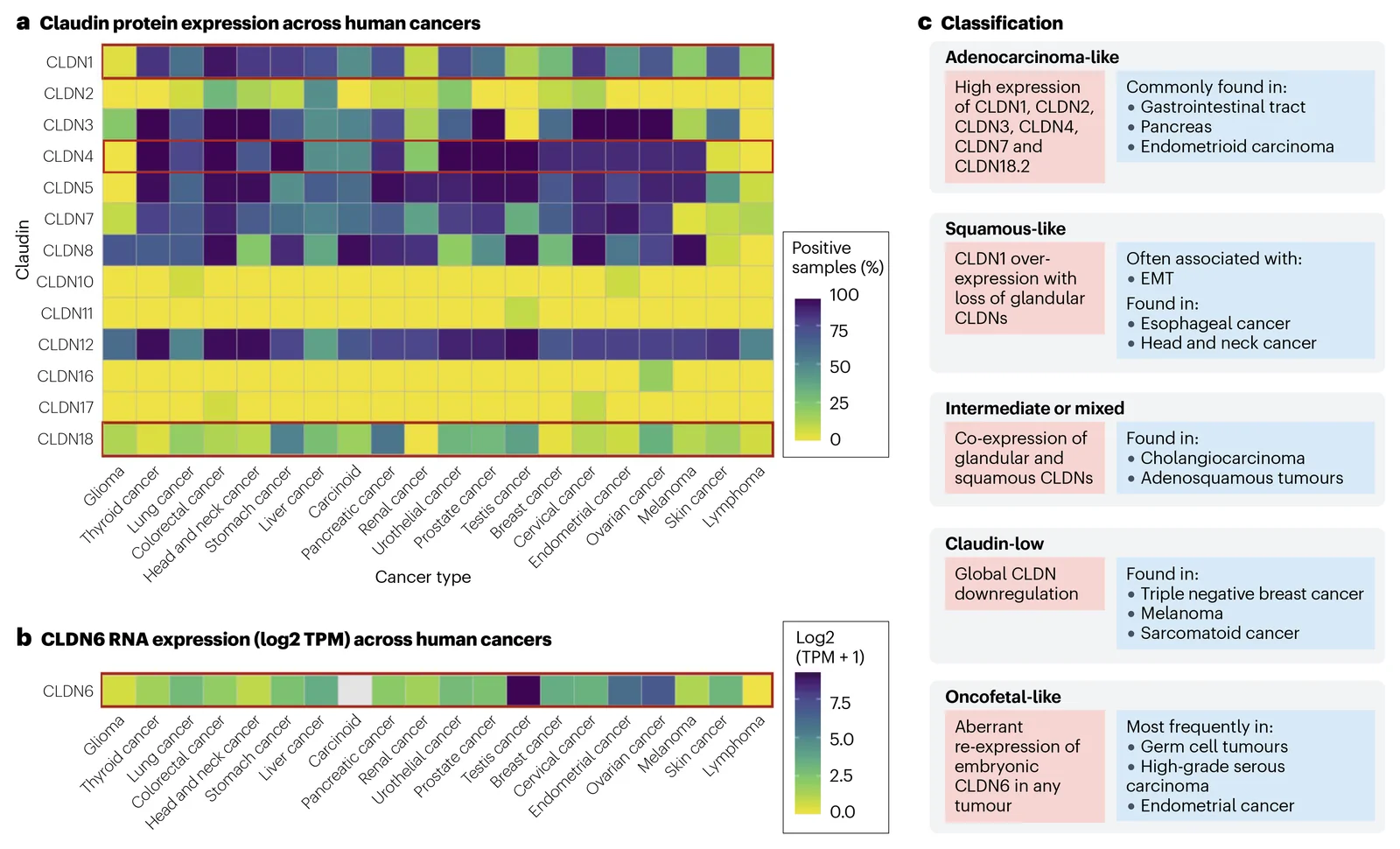
CLDN expression across human cancers and associated therapeutic relevance. a. Prevalence of CLDN protein positivity across major cancer types based on immunohistochemistry (IHC) data from the Human Protein Atlas version 24 (HPA v24)^254^ The most clinically advanced targets are highlighted by a red border. Expression data indicate the proportion of tumor samples annotated as positive (defined by HPA as low, medium, or high IHC staining), across all available cases for each cancer type in the database. When more than one validated antibody was available for a given CLDN, the percentage values shown reflect the average across all antibodies. For CLDN18, the antibodies used detect both CLDN18.1 and CLDN18.2 isoforms and do not allow isoform-specific quantification. b. RNA expression levels of CLDN6 across cancer types, shown as log_2_-transformed transcript per million (TPM) values from The Cancer Genome Atlas (TCGA) program accessed through the HPA v24. As protein-level data were not available for CLDN6, transcript abundance is shown to reflect tissue specificity. RNA levels were downloaded as TPM and log_2_(TPM + 1) values were calculated without additional normalization. When multiple tumor subtypes were listed for the same organ, their values were averaged to mirror the categorization used for protein-level data. c. Proposed classification of CLDN tumor-expression patterns. This author-defined framework is derived from a synthesis of Clinical Proteomic Tumor Analysis Consortium (CPTAC) mass spectrometry-based proteomics data, accessed via the HPA, in conjunction with protein IHC expression data (panel a) and RNA expression profiles from TCGA, and relevant literature. The framework categorizes tumors into recurring CLDN expression patterns: adenocarcinoma-like, squamous-like, intermediate/mixed, claudin-low, and oncofetal-like.

Because transcript levels, protein overexpression, and membrane deregulation often show non-linear or discordant correlations when analyzing CLDNs, semi-quantitative immunohistochemistry (IHC) remains the most clinically informative approach for CLDN quantification^19,40,41^. However, available IHC data do not systematically resolve subcellular distribution, particularly membrane localization, thereby limiting the ability to make confident inference about cell-surface accessibility. An overview of CLDN protein expression patterns across solid tumors is presented in Fig. 2 and Table 2. Although available databases (e. g. the Human Protein Atlas, TCPA portal) provide a useful reference for CLDN detection across tumor types, their accuracy may be limited by insufficient resolution or the small size of patient cohorts. As such, these resources may under- or misestimate CLDN prevalence.

Among all CLDNs, expression of the CLDN18.2 isoform is the most extensively studied. It is expressed in approximately 30–40% of esophageal adenocarcinomas and gastric cancers but is largely absent in CRC, except in select molecular subtypes such as microsatellite instability-high (MSI-high) and mucinous tumors^42,43^. CLDN1 is frequently overexpressed across several tumor types, including colorectal adenocarcinoma, esophageal squamous cell carcinoma (SCC), head and neck SCC (HNSCC), and hepatobiliary cancers^33,34,44–48^. CLDN4 shows consistently high expression in gastrointestinal, pulmonary, hepatobiliary and ovarian 6 tumors^46,49–52^. Based primarily on transcriptomic analyses, CLDN6 expression is highly prevalent in testicular germ-cell tumors and is also observed in aggressive subtypes of ovarian and endometrial cancers^53–55^. CLDN3 and CLDN7 are commonly overexpressed in gynecologic malignancies, prostate cancers, and gastrointestinal adenocarcinomas^25,31,52,56^.

Across solid tumors, CLDNs can exhibit intratumor heterogeneity. For example, studies on CLDN18.2 in gastric cancers have demonstrated intratumor heterogeneity in approximately 60% of cases^57,58^. Moreover, primary and metastatic cancer tissues can differ, with up to 25% of cases showing discordance in CLDN18.2 expression^57,59^. Among various metastatic organ sites, peritoneal lesions exhibited the highest CLDN18.2 positivity rate (44.3%) and positive concordance rate (31.4%), whereas liver lesions showed the lowest positivity rate (17.9%) and concordance rate (12.8%)^57^.

Beyond organ-specific expression patterns, CLDN expression across human tumors can reflect underlying epithelial reprogramming. Based on an integrative analysis of CLDN expression patterns across tumor types, together with associations reported in the literature, we propose five broad categories relating CLDN expression to functional associations (Fig. 2)

Finally, it is important to note that CLDN expression may be dependent on tumor grade and differentiation but may also contribute to these features through its roles in epithelial polarity, adhesion and signaling. Accordingly, poorly differentiated tumors may ultimately lose the expression of epithelial markers, including CLDNs^60–62^.

### Mechanisms of CLDN deregulation in cancer

The mechanisms underlying CLDN dysregulation have been studied in some cancers but are not completely elucidated in all cases. Several tissue and tumor-dependent mechanisms at the genetic, epigenetic, transcriptional, and post-translational levels have been identified.

#### Genetic alterations

To obtain an overview on CLDN genetic alterations in cancer, we analyzed data from The Cancer Genome Atlas (TCGA) using the cBioPortal for Cancer Genomics. This analysis indicates that CLDN genes can be altered across multiple tumor types, with copy number alterations occurring far more frequently than point mutations^63–66^. The highest frequencies of CLDN alterations are observed in squamous cell carcinomas (SCC), including lung, esophageal, head and neck, cervical, and bladder cancers, but are also seen in certain adenocarcinomas such as ovarian and gastric cancers, where up to 40–60% of tumors harbor alterations in at least one CLDN gene, most commonly copy number amplifications (CNAs)^63–66^. In contrast, hematological malignancies, kidney cancers, and thyroid carcinoma display low alteration frequencies. However, overall, genetic alterations across the *CLDN* family are generally infrequent, with *CLDN1, CLDN11*, and *CLDN16* being the most commonly affected^63–66^. In lung SCC, up to one-third of cases exhibit genetic alterations in these three genes^63–66^.

CLDN1 genetic alterations are very frequent in SCC, particularly of the lung, esophagus, head and neck, cervix, and bladder, where approximately 20–30% of tumors are affected, predominantly through CNAs. In adenocarcinomas, CLDN1 alterations occur at lower frequencies, with the highest rates observed in esophageal and ovarian adenocarcinomas (about 10–15%), whereas most other adenocarcinomas types show alterations in 5% or fewer tumors^63–66^.

CLDN4 and CLDN6 alterations, mostly CNAs, are relatively rare across cancers, generally affecting less than 4% of tumors. CLDN4 alterations are more common in epithelial adenocarcinomas, particularly of the stomach, ovary, and endometrium. CLDN6 alterations are preferentially enriched in breast, stomach, and cholangiocarcinoma.

CLDN18 alterations are most frequent in SCCs of the lung, cervix, and head and neck, with a prevalence of 5–10%. Among adenocarcinomas, the highest rates are found in esophageal adenocarcinoma (~8%), followed by endometrial, ovarian, and gastric cancers (~5–6%). While CNAs are the predominant alteration, gastric adenocarcinoma uniquely shows a notable enrichment in structural variants, present in 2.8% of cases^63–66^.

Conversely, *CLDN22, CLDN23*, and *CLDN24* exhibit mostly deep deletions, suggesting potential tumor suppressor roles^63–67^.

Another rare genetic event observed across various tumor types are fusion events involving *CLDN* genes. A notable example is the fusion of *CLDN18* to Rho GTPase-activating protein 26 (*ARHGAP26)*, primarily detected in gastric adenocarcinomas with a prevalence of approximately 3% and up to 17% in signet-ring cell carcinoma **[G]**^63–65,68^. This rearrangement, which results in the truncation of CLDN18 and fusion to the RhoGAP domain of ARHGAP26, is known to promote epithelial barrier disruption and oncogenic Yes-associated protein (YAP)–transcriptional co-activator with PDZ-binding motif (TAZ) signaling, supporting its role as a potential driver event and therapeutic target, particularly in gastroesophageal cancers^69^. Beyond *CLDN18*, several other *CLDN* family members are involved in fusion events with diverse partners. For example, CLDN14 fusions of unknown significance with C21orf59, NRAS, or RUNX1 have been observed^63–66^. Rare fusions of unknown significance involving *CLDN1, CLDN10, CLDN11*, and *CLDN7* have been sequenced^63–66^. Most of these fusions are isolated occurrences, and many lack functional annotation or experimental validation. The directionality of these events (whether *CLDN* is the 5′ or 3′ fusion partner) varies, suggesting that both gain- and loss-of-function mechanisms may be at play. Importantly, none of the fusions outside *CLDN18* occurred at a frequency sufficient to suggest recurrent oncogenic drivers; however, their presence across multiple cancers underscores the broader potential of *CLDN* family members to undergo gene rearrangements.

Overall, these data indicate that *CLDN* genetic alterations are likely not major oncogenic drivers. Mutations across the *CLDN* gene family are rare, typically of uncertain clinical significance, and there is no evidence of recurrent hotspot mutations to date^63–66^. The most commonly observed alterations are CNAs, more frequently observed in SCC and generally less common in adenocarcinomas. However, because CLDN amplifications are overall infrequent, CNAs alone are unlikely to account for the widespread CLDN overexpression observed across tumor types, implying a major contribution of transcriptional, epigenetic and/or post-transcriptional mechanisms. Among the CLDN gene family, gene fusions appear to be of functional relevance mainly for CLDN18 in gastric cancer.

#### Transcriptional regulation

Environmental factors associated with cancer, including pro-inflammatory signals and hypoxia have been shown to modulate CLDN expression. Pro-inflammatory signaling is a major driver: tumor necrosis factor (TNF) induces expression of multiple CLDNs, most notably CLDN1, through mitogen-activated protein kinases (MAPK) or nuclear factor kappa-B (NF-κB) transcriptional activation^16,70^. This TNF-driven CLDN1 upregulation is well documented in liver and CRC^16,71^.

Hypoxia provides a second, recurrent driver. Through hypoxia-inducible factor 1α (HIF-1α) activation, hypoxic stress induces CLDN expression in multiple epithelial cancers. Example include HIF-1α–dependent induction of CLDN1 and CLDN4 in esophageal SCC^72^ or cyclic hypoxia **[G]–**driven upregulation of CLDN7 in nasopharyngeal carcinoma^73^.

Growth factor signaling also modulates CLDN transcription, particularly via transforming growth factor-β (TGFβ). The canonical SMAD pathway can modulate CLDN expression^74^ as suggested by altered intestinal claudin patterns after *Smad4* deletion in mice with increased Cldn2 and Cldn8 and decreased Cldn3, Cldn4 and Cldn7^75^. In lung cancer cell lines, TGFβ induces CLDN4 through convergent SMAD-dependent signaling and a JUN-dependent promoter activation, and CLDN4 is functionally required for TGFβ-driven migration and anchorage-independent growth in standard in vitro assays^76^. TGFβ-associated CLDN4 upregulation has also been reported in glioblastoma and ovarian cancer ^77,78^. By contrast, in benign prostatic epithelial cells, TGFβ1 downregulates CLDN1 via non-canonical MEK1/2– ERK1/2 signaling, and MEK inhibition restores CLDN1 expression^79^.

These external signals intersect with lineage and epithelial–mesenchymal transition **[G]** (EMT)-associated transcriptional control. As an example, in gastric cancer, promoter bioinformatic analyses implicate epithelial differentiation and proliferation regulators (GATA4, GATA6, Krüppel-like factor 5) together with inflammatory and stress-responsive factors (NF-κB and activator protein 1 (AP-1) family members) in CLDN9 regulation^80^. During EMT, EMT transcription factors can directly reprogram CLDN expression, exemplified by TWIST1-driven activation of CLDN4 transcription in cervical cancer, which promotes migration and invasion^81^.

Finally, nuclear hormone signaling contributes to CLDN regulation. In breast cancer cells, 17β-estradiol upregulates CLDN6 via estrogen receptor α (ERα) signaling^82^. while in endometrial cancer CLDN6 can activate ERα in a ligand-independent manner through SRC–PI3K–AKT–SGK–mediated phosphorylation of ERα at Ser518^83^.

#### Epigenetic regulation

Promoter hypermethylation is associated with claudin silencing in several settings, including CLDN10B in renal cancers, where hypermethylation correlates with aggressive disease and poor prognosis^84^. Supporting a causal link, CRISPR–dCas9-mediated promoter demethylation in a human embryonic cell line increases endogenous CLDN10B expression and reduces migration and spheroid formation^84^.

Similar repressive mechanisms have been reported for other claudins: in ovarian cancer, CLDN4 is negatively regulated by promoter methylation and histone H3 deacetylation^85^ and in gastric cancer, promoter methylation downregulates CLDN4 and CLDN9 ref^80,86^. More broadly, breast cancers - especially the CLDN-low breast cancer **[G]** subtype - show global aberrant DNA hypermethylation that may contribute to the phenotype ^87^. Epigenetic control can also increase claudin expression: in cholangiocarcinoma, the demethylase TET1 promotes CLDN3 transcription through promoter demethylation, modulated by the phosphatase PPM1G^88^. In addition to DNA methylation, histone deacetylation can repress CLDNs, as described for CLDN1 in colon cancer^89,90^.

Overall, these studies indicate that promoter methylation and histone deacetylation contribute to dysregulated CLDN expression in cancer, although their relative frequency and impact compared with other regulatory mechanisms remains to be determined.

## Functional role of altered CLDN expression in cancer

**Figure 3:**
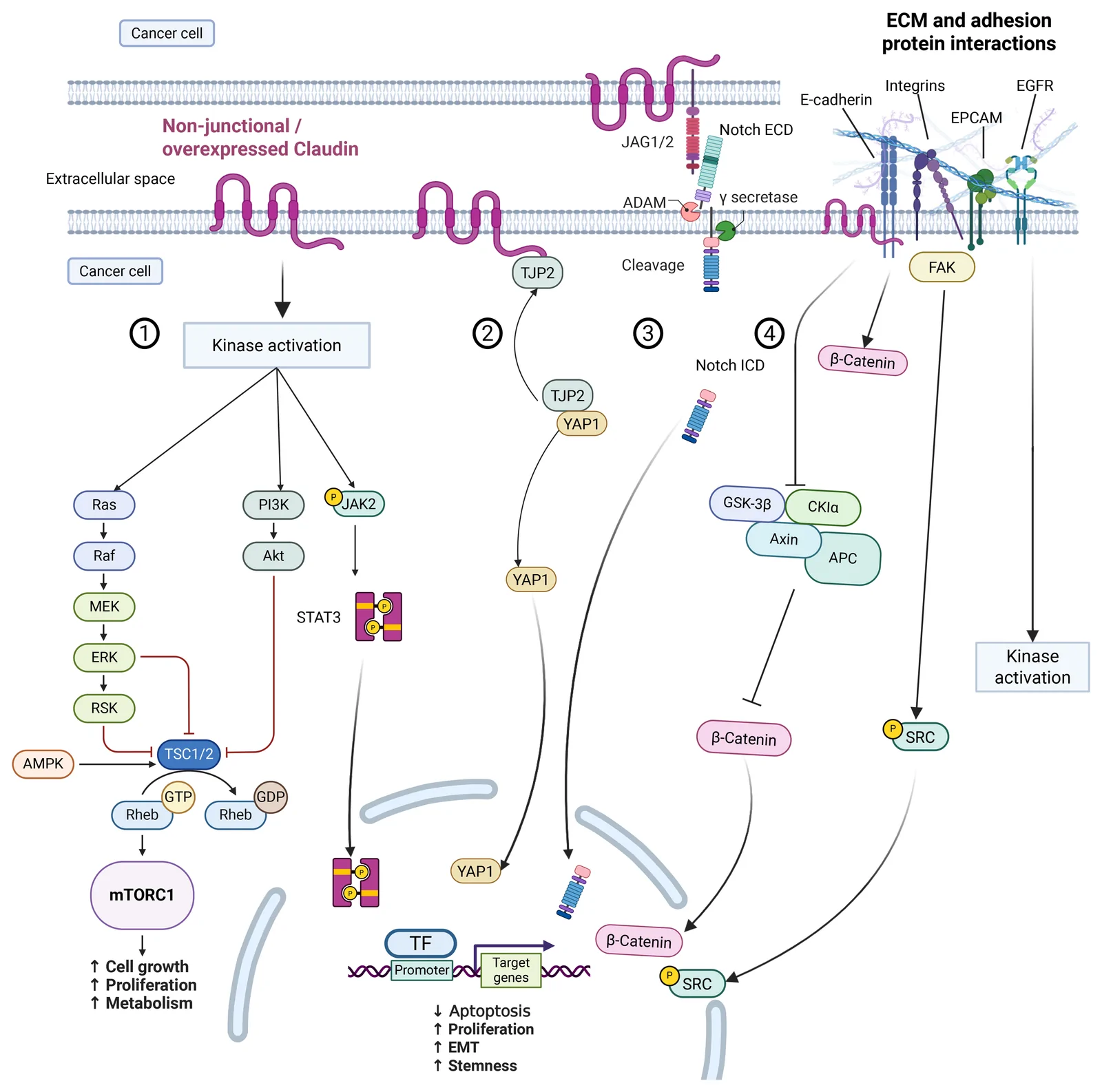
Examples for oncogenic signaling pathways activated by CLDNs in cancer. Claudins (CLDNs), when overexpressed or mislocalized to non-junctional regions of the cell membrane, can initiate, modulate and amplify multiple oncogenic signaling pathways. Non-junctional CLDNs lose their normal barrier-forming functions and instead participate in aberrant signal transduction events that contribute to tumor progression. **a**. CLDNs promote the activation of oncogenic kinase pathways: Examples are: the RAS– RAF–MEK–ERK mitogen-activated protein kinase (MAPK) pathway, supporting proliferation and transcriptional activation via extracellular signal-regulated kinase (ERK) and ribosomal S6 kinase (RSK). The PI3K–AKT–mechanistic target of rapamycin complex 1 (mTORC1) pathway, where PI3K activates AKT, which inhibits the tuberous sclerosis complex (TSC1/2), leading to Ras homolog enriched in brain (RHEB)-mediated activation of mTORC1, and promoting cell growth and metabolism. The Janus kinase 2 (JAK2)– signal transducer and activator of transcription 3 (STAT3) axis, in which JAK2 phosphorylates STAT3, driving transcription of anti-apoptotic and pro-proliferative genes. **b**. Yes-associated protein 1 (YAP1) is activated upon CLDN overexpression. In normal cells, YAP1 is retained in the cytoplasm through its interaction with tight junction protein 2 (TJP2). Upon CLDN overexpression, TJP2 is sequestered away from YAP1, allowing YAP1 to translocate into the nucleus and activate transcriptional programs that enhance proliferation, survival, epithelial-to-mesenchymal transition (EMT), and stemness. **c**. CLDNs at the membrane can interact with Jagged ligands (JAG1/2) on neighboring cells, promoting NOTCH receptor cleavage by a disintegrin and metalloproteinase domain-containing protein (ADAM) proteases and γ-secretase. The NOTCH intracellular domain (ICD) is released and translocates into the nucleus, where it activates target genes involved in cell fate specification and stem-like behavior. **d**. CLDNs associate with and modulate the function of adhesion proteins such as E-cadherin, EpCAM, and integrins, promoting downstream activation of focal adhesion kinase (FAK) and SRC-family kinases, as well as other pathways like MAPK. Additionally, CLDNs interfere with the β-catenin destruction complex (comprising APC, Axin, GSK-3β, and Casein kinase I alpha (CKIα)), leading to β-catenin stabilization and nuclear translocation, reinforcing WNT-like transcriptional activation. These coordinated signaling events promote hallmark cancer traits, including increased proliferation, loss of polarity, EMT, and acquisition of stemness.

A number of CLDNs have been implicated into a variety of tumor-related processes (Fig. 3) while others appear to serve primarily as lineage-restricted tumor markers.

Importantly, our understanding of CLDNs in cancer biology is incomplete. Much of the current evidence is derived from cell lines, organoids, or transcriptomic associations and downstream signaling cascades for many CLDNs are only partially understood. Deepening the biologic understanding and deciphering the context-dependent function of CLDNs is an important future goal to enable more accurate therapeutic targeting and the development of rational combination strategies. Most CLDN studies have been carried out in cancer cell lines, but the expression and function of CLDNs, as either tumor promoters or suppressors, can vary widely depending on the type of cancer, the experimental model, and the specific cell line used (see Table 2). For instance, CLDN6 inhibits the growth and metastatic potential of breast cancer cells, whereas CLDN6 supports the proliferative and invasive phenotypes in hepatocellular carcinoma (HCC) and gastric cancer^17,83,91–93^. Similarly, loss of CLDN7 promotes stemness and metastatic features in colorectal cancer models, whereas in pancreatic cancer CLDN7-EpCAM interactions have been showed to drive enhanced migration, proliferation, apoptosis resistance in rat tumor cell line^94–96^. Additionally, mechanistic studies are sometimes performed in cancer models that do not correspond to the tumor types exhibiting the highest levels of CLDN expression. Moreover, although the majority of available data supports a tumor-promoting role for CLDNs (summarized in Table 2), this may partly reflect a research bias, as such mechanisms are often prioritized due to their potential to reveal actionable or targetable pathways.

In the following sections, we will explore examples of how CLDNs shape cancer biology through their functional roles.

### Tumor growth and signaling

CLDN protein overexpression has been shown to induce oncogenic signaling resulting in enhanced tumor growth. CLDN1 is among the most extensively studied CLDN and activates PI3K–AKT–mTOR, NOTCH1 and MAPK–ERK signaling in hepatobiliary, breast, oral, and lung cancers^33,97–99^. For hepatocellular carcinoma (HCC), cholangiocarcinoma and CRC, genetic CLDN1 gain- and loss of function studies in cell-based and in vivo models showed a robust and significant effect on tumor growth suggesting a driver role of CLDN1 in these tumors^32,33,100^. In CRC cell lines, mechanistic perturbation experiments indicate that CLDN1 binds and stabilizes ephrin type-A receptor 2 (EPHA2) via its PDZ-binding motif, sustaining AKT–CD44 signaling and stemness^98^. CLDN1 can also act downstream of the RNA-binding protein LIN28B, which post-transcriptionally increases CLDN1 mRNA and engages NOTCH3-dependent signaling, promoting invasion and liver metastasis in mouse models^101^; consistent with this pro-growth role, a CLDN1 mAb exerts a cytostatic effect in colon cancer spheroids^102^. In preclinical liver cancer models, antibody-mediated targeting of CLDN1 disrupts signaling of multiple kinases implicated in liver carcinogenesis - including NOTCH1, epidermal growth factor receptor (EGFR), SRC, vascular endothelial growth factor receptor (VEGFR) and members of the AKT family^16,33^. Moreover, treatment with CLDN1 mAbs suppress tumor growth and proliferation in HCC mouse models and human spheroids^33^. In other tumor types additional pathways have been implicated: in an oral squamous carcinoma cell line, integrative analyses associate CLDN1 with PI3K–AKT–mTOR signaling, although without definitive mechanistic validation^99^; in breast cancer, disintegrin and metalloproteinase domain-containing protein 15 (ADAM15) upregulates CLDN1 and activates PI3K–AKT–mTOR, reinforcing proliferative programs^97^; and in human cervical cancer cell lines, CLDN1 contributes to proliferation and migration, while CLDN1 inhibition disrupts cell-cycle progression and promotes apoptosis^103^.

Beyond CLDN1, CRC models support growth-promoting roles for additional CLDNs that converge on EGFR–ERK and PI3K signaling. CLDN2 overexpression in CRC cell lines enhanced proliferation, anchorage-independent growth, and tumorigenicity in xenograft mouse models. This phenotype depends on EGFR signaling via ERK1, ERK2 and PI3K pathways, further supporting the role of CLDN2 as a growth driver in colon cancer^104^. Similarly, CLDN8 14 overexpression promotes proliferation, migration and invasion via MAPK–ERK activation and matrix metalloproteinase 9 (MMP9) induction, while ERK inhibition or CLDN8 knockdown suppresses these phenotypes^105^.

Another signaling pathway is Hippo–YAP signaling, which also illustrates the context dependence. In diffuse gastric cancer, the CLDN18–ARHGAP26 fusion induces signet-ring phenotypes via Ras homolog family member A (RHOA)– focal adhesion kinase (FAK)–YAP activation with FAK and YAP–TEAD inhibition significantly reducing tumor progression in *Trp53*-deficient murine organoids and xenografts^69^. Conversely, CLDN18.1 appears tumor-suppressive, as its loss leads to YAP–TAZ activation and spontaneous lung adenocarcinoma in knockout mice^106^. CLDN6 also engages Hippo–YAP signaling with context-dependent roles: it drives EMT in gastric cancer^93^ and sorafenib resistance in HCC^17^ yet appears tumor-suppressive in breast and lung cancer cell lines and xenografts by inducing apoptosis through activation of the receptor-interacting protein kinase 1 (RIP1)–ASK1–p38–JNK pathway^107,108^. Collectively, these data illustrate the relevance of cellular context in CLDN-mediated effects on tumor growth and proliferation.

### EMT and metastasis

Across cancers, CLDNs influence EMT and metastasis by activating signaling pathways that regulate cell invasion, **anoikis [G]**, and extracellular matrix remodeling. In hepatobiliary and gastrointestinal cancer cell models, CLDN1 is predominantly pro-metastatic: in HCC cell lines, it induces EMT and invasion via ABL–ERK and NOTCH1 signaling^33,100,109^ and promotes matrix remodeling through an ABL–protein kinase C δ (PKCδ)–MMP2 axis^110^.

In CRC and gastric cancer, evidence from cell lines and xenografts suggest that CLDN1 further facilitates metastatic dissemination by enhancing resistance to anoikis. In CRC, this 15 occurs via recruitment of SRC and ZO1, leading to activation of the SRC–AKT–BCL2 survival pathway^111^. In gastric cancer, CLDN1 supports survival during cell detachment by stabilizing membrane β-catenin and sustaining SRC–AKT signaling^112^. Moreover, in CRC cell line-derived models, CLDN1 promotes EMT and metastatic traits by downregulating E-cadherin and enhancing β-catenin/TCF signaling^32^. CLDN1 also mediates TNF-induced EMT through activation of ERK1, ERK2 and SRC in CRC cell lines^71^. However, in human lung adenocarcinoma cell lines, CLDN1 has been shown to suppress EMT by upregulating ephrin type-B receptor 6, inhibiting ERK signaling, and reducing SLUG expression^113^.

Other CLDN family members promote EMT through distinct mechanisms. CLDN2 contributes to invasion and metastasis via the ZO1–ZONAB complex, which represses N-MYC downstream-regulated gene 1 (NDRG1) in CRC cell lines^27^ and in breast cancer cell lines, through its PDZ domain, which interacts with the actin-binding scaffold protein Afadin (also known as AF6)^114^. In gastric cancer cell-derived models, CLDN6 promotes EMT by interacting with large tumor suppressor kinases 1 and 2 (LATS1 and LATS2), thereby inhibiting YAP1 phosphorylation and enhancing YAP1 nuclear activity, which induces SNAIL expression^93^. CLDN11 promotes migration and lymphatic dissemination in HNSCC cell lines recruiting SRC to activate p190RhoGAP, thus suppressing RhoA activity at junctions and facilitating coordinated migration^115^; in small-cell lung cancer, it cooperates with membrane type 1-matrix metalloproteinase (MT1-MMP, also known as MMP14) to enhance pro-MMP2 activation^116^.

CLDN3 and CLDN4 exhibit an inverse relationship with EMT in ovarian cancer cell lines: knockdown of either protein reduces E-cadherin expression and activates β-catenin/TCF and PI3K–AKT signaling, thereby enhancing invasion and metastasis^117,118^.

### Cell plasticity, stemness and differentiation

Multiple studies demonstrated that CLDNs regulate epithelial differentiation, stemness, and plasticity **[G]** of cancer, often by engaging developmental pathways that control progenitor-like states. In HCC, CLDN1 overexpression in cell-based models promotes stemness by activating NOTCH through juxtacrine engagement of Jagged 1 (JAG1). Gain- and loss-of-function studies show that CLDN1 modulates NOTCH1 signaling enhancing tumor growth and progenitor marker expression^33^.

CLDN7 illustrates how CLDN functions can diverge between normal tissue homeostasis and cancer. In normal colon, CLDN7 is low in stem and progenitor cells and higher in differentiated cells, and this pattern is regulated by WNT–TCF4 through SOX9-mediated repression of CLDN7^119^. Despite this ´differentiation-associated´ expression pattern, genetic loss-of-function studies show that CLDN7 is required to maintain intestinal stem cell integrity in vivo. In mice with inducible *Cldn7* knockout, loss of CLDN7 depletes active intestinal epithelial stem cells, drives aberrant villus proliferation, and impairs multilineage differentiation^120^.

In CRC, CLDN7 effects appear strongly context dependent. Forced CLDN7 expression in polarized HT29 3D spheroids disrupts epithelial polarity, increases TCF4 target gene expression, accelerates spheroid growth, and enhances xenograft tumorigenicity; conversely, SOX9 induction in HT-29Cl.16E cells suppresses CLDN7 and restores junctional integrity^119^.

In contrast, in HCT116 CRC cells, CLDN7 depletion can enrich stem-like behavior. Stable CLDN7 knockdown in CD133^+^CD44^+^ subpopulations increases proliferation, migration, and xenograft growth while reducing apoptosis, and inducible intestinal-specific *Cldn7* knockout in C57BL/6 mice produces adenomas and altered crypt differentiation^95^. Collectively, these opposing outcomes suggest that CLDN7-dependent regulation of stemness and differentiation is shaped by lineage, subtype, and experimental context.

CLDN2 has also been implicated in regulating plasticity in CRC. Overexpression of CLDN2 promotes spheroids self-renewal, enriches aldehyde dehydrogenase (ALDH)^high^ populations, facilitates initiation and metastasis, and supports post-chemotherapy recurrence across patient-derived organoids, cell lines, and xenografts. Mechanistically, CLDN2 activates YAP and suppresses miR-222-3p to promote stemness^121^.

### Therapy resistance

The overexpression of several CLDNs has been shown to be functionally associated with therapy resistance across cancer types by sustaining pro-survival signaling, restricting drug penetration, and/or promoting phenotypic plasticity. In CRC, CLDN1 is a treatment-inducible resistance factor. Chemotherapy, particularly oxaliplatin, induces CLDN1 through a MAPK–p38–glycogen synthase kinase 3 β (GSK3β)–WNT–β-catenin axis, in which p38 inactivates GSK3β, stabilizes β-catenin, and transcriptionally upregulates CLDN1 ref^122^. Pharmacologic inhibition of p38 or WNT–β-catenin, or GSK3β knockdown, blocks CLDN1 induction, and CLDN1 silencing restores drug sensitivity and apoptosis in oxaliplatin-resistant CRC cell-line models^122^. An anti-CLDN1–monomethyl Auristatin E (MMAE) antibody drug conjugate (ADC) after an oxaliplatin treatment course yields synergy in 3D cultures and significantly inhibits xenograft growth when given to tumor-bearing mice^122^.

Beyond transcriptional induction, CLDNs can also modulate drug response by altering permeability and stress responses. In non-small-cell lung cancer, CLDN1 increases doxorubicin tolerance in human lung squamous cell carcinoma cell lines and 3D spheroids by reducing paracellular drug permeability via PI3K–AKT–NF-κB signaling^123^. CLDN2 has a similar effect in lung adenocarcinoma cell lines, where overexpression reduces intracellular accumulation of agents such as doxorubicin^124^ and activates nuclear factor erythroid 2–related factor 2 (NRF2)-mediated antioxidant signaling via glucose-transport inhibition^124,125^. Similarly, in epithelial ovarian cancer, CLDN4 facilitates cisplatin resistance by limiting its intracellular uptake^126,127^.

A mechanistically distinct resistance mechanism is described in HCC, where CLDN6 promotes sorafenib resistance by disrupting Hippo–YAP signaling. Tight junction protein 2 (TJP2) normally binds YAP1 and retains it in the cytoplasm for degradation. CLDN6 competes with YAP1 for TJP2 binding, displacing YAP1 and enabling its nuclear translocation. This stabilizes YAP1 and activates Hippo–YAP signaling, driving expression of multidrug-resistance genes^17^. CLDN6 overexpression reduces sorafenib sensitivity in hepatocellular carcinoma cell lines and xenograft mouse models, while its knockdown restores drug sensitivity and reduces colony formation, proliferation, and sphere-forming ability in cancer cell lines, and in xenografts it markedly suppresses tumor growth^17^.

### Tumor microenvironment interactions

While the role of CLDNs in the epithelial cancer biology has been described in great detail, the functional role of CLDNs to shape stromal dynamics and immune infiltration within the TME is currently emerging. An example is the pathogenic role of CLDN1 in fibrosis-driven liver disease and cancer models: In mouse models of liver fibrosis and carcinogenesis, genetic knockdown or antibody-mediated inhibition of CLDN1 reduces fibrotic remodeling and tumor occurrence in the liver, suggesting that CLDN1 contributes to stromal activation and early tumor-promoting processes in diseased liver tissue^16^. Similarly, treatment with CLDN1 mAb has been shown to robustly reduce biliary fibrosis **[G]** across several mouse models for biliary fibrosis and cholangiopathies **[G]** ^128^. Moreover, in patient-derived tumor spheroids treatment with CLDN1 mAb resulted in reprogramming of the TME, including increased CD8^+^ T cell effector function, suggesting a role for CLDN1 in the tumor immune environment^33^. These data provide opportunities to treat fibrosis-associated tumors such as HCC, cholangiocarcinoma, pancreatic adenocarcinoma and head and neck cancer or combine CLDN1-targeting therapies with immune checkpoint inhibitors **[G]**^33^.

Spatial profiling studies in gastric cancer suggest that CLDN18.2 expression is associated with a distinct immune architecture. Compared to CLDN18.2-negative gastric cancers, CLDN18.2-positive tumors exhibit a higher density of CD4^+^ and CD8^+^ T cells that are largely non-exhausted and positioned closer to tumor cells, as well as increased neutrophil presence. Despite this apparent immune activity, CLDN18.2-positive tumors are associated with worse prognosis, suggesting that immune infiltration does not translate into effective checkpoint-mediated control^129,130^. Associations with programmed cell death protein 1 (PD1) and Programmed Death-Ligand 1 (PDL1) vary across datasets. As an example, one study reports ~17% PDL1 positivity (combine positive score **[G]** (CPS) of at least 5) in tumors with CLDN18.2 expression in at least 70% of cells^131^, whereas another reports approximately 40% PDL1 (CPS of at least 5) among CLDN18.2-positive tumors, which is lower than the 50-60% observed in CLDN18.2-negative tumors^24,132^. Interestingly, neoadjuvant chemotherapy can further reshape the immune landscape of CLDN18.2-positive tumors by increasing the number of regulatory T (T_reg_) cells, tumor-associated macrophages (TAMs), and natural killer (NK) cells, with dampened NK cytotoxic programs and enhanced immunosuppressive signaling via TGFβ and the C-C motif chemokine ligand 5 (CCL5)– C-C motif chemokine receptor 5 (CCR5) axis^133^.

Similar to CLDN18.2, CLDN9 has emerged as a prognostic TME marker in gastric cancer: integrated transcriptomic analysis of TCGA and GEO datasets identified a TME high risk signature for poor prognosis cancers including CLDN9 ref^134^. In glioblastoma, CLDN14 appears to play a role in vascular integrity and immune infiltration^135^: PAK4-mediated suppression of CLDN14 promotes mesenchymal-like reprogramming of tumor-associated endothelial cells, leading to leaky vasculature and reduced T-cell adhesion, whereas PAK4 inhibition restores CLDN14, normalizes vasculature, increases T-cell infiltration, and improves response to chimeric antigen receptor^135^.

Collectively, the current data suggest that CLDN expression in cancer cells may influence the TME by modulating fibrosis, immune infiltration, stromal interactions, and vascular integrity, but definitive causal roles—and the most actionable therapeutic entry points—will require further mechanistic and clinical validation.

## Clinical Development of CLDN-targeted therapies

Among the CLDN family members, current clinical development is focused primarily on CLDN1, CLDN6, CLDN4 and CLDN18.2, utilizing a variety of platforms including monoclonal mAbs, ADCs, bispecific and trispecific antibodies or CAR T cells. While clinical development is most advanced for CLDN18.2 in gastroesophageal and gastric adenocarcinomas, the broader objective is to expand these therapies toward other tumor types that express CLDNs in a restricted or tumor-specific manner, mirroring the tumor-agnostic paradigm seen with other targeted therapies.

**Figure 4:**
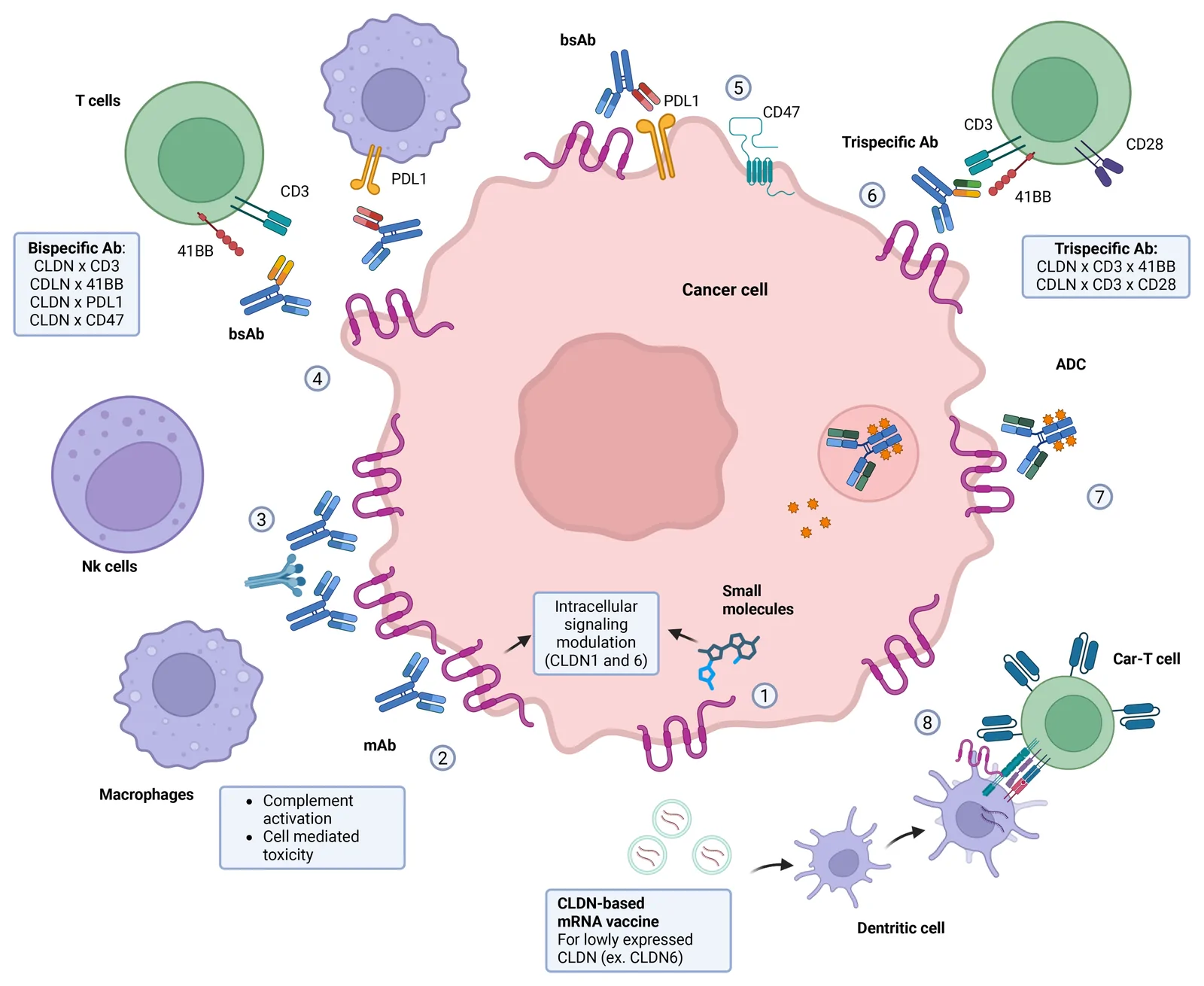
Therapeutic modalities for CLDN-targeted therapy. Schematic overview and examples of distinct CLDN-directed formats to target cancer cells. CLDN-targeted therapies can comprise intracellular small molecule inhibitors that interact with the PDZ domain to modulate intracellular signaling. Monoclonal antibodies (mAb) can be used to modulate intracellular signaling and/or to engage Fc-dependent effector functions, including, antibody-dependent cellular cytotoxicity, antibody-dependent cellular phagocytosis and complement-dependent cytotoxicity. Bispecific antibodies can co-target CLDN on tumor cells and a receptor on T cells or antigen-presenting cells to promote T cell engagement and activation; another modality is to co-target CLDN and programmed death-ligand 1 (PDL1) on tumor cells to couple tumor targeting with checkpoint blockade and improve immune response. Trispecific antibodies can be used to target CLDN on tumor cells together with two receptors on T cells to enhance T cell engagement and prime activation. Antibody–drug conjugates (ADCs) bind CLDN on tumor cells, internalize, and release a cytotoxic payload to induce cell death. CLDN-directed chimeric antigen receptor (CAR) T cells can recognize CLDN and mediate cytotoxicity; their activity depends on antigen density, accessibility, and spatial distribution. CLDN mRNA vaccines (CARVac) can be combined to prime or boost CLDN-specific CAR T cell responses for lowly-expressed CLDNs, such as CLDN6.

Therapeutic strategies can be broadly categorized based on whether they exploit accessible, non-junctional CLDN overexpression on the cell surface to mediate immune or cytotoxic killing, for example, mAbs, ADCs, bispecifics, CAR T cells targeting for example CLDN18.2 ref^136^ (Fig. 4), or whether they target CLDN-dependent intracellular signaling pathways involved in tumor progression, such as EMT, MAPK–ERK activation, and stemness (for example, PDZ domain inhibitors targeting CLDN1 ref^137^). A third category combines both approaches. Examples of this combined strategy are anti-CLDN1 ref^33^ and CLDN6 mAbs^17^, which not only recognize tumor cells via elevated CLDN1 and CLDN6 expression but also inhibit downstream oncogenic intracellular signaling pathways. Although agents targeting the intracellular domains of CLDNs are still in preclinical development, they are emerging as a complementary therapeutic strategy. Below, we summarize therapeutic advances by targeted CLDN, with further details on clinical-stage agents and preclinical or investigational new drug (IND)-enabling agents provided in Supplementary Tables 1 and 2, respectively.

### CLDN18.2 at the forefront of CLDN-directed therapies

CLDN18.2-targeted therapies have pioneered the development of CLDN-directed drugs. Zolbetuximab, which induces antibody-dependent cell-mediated cytotoxicity **[G]** (ADCC) and complement-dependent cytotoxicity **[G]**, has shown improved progression-free survival (PFS) and overall survival (OS) when combined with chemotherapy in patients with CLDN18.2-positive gastroesophageal adenocarcinoma, leading to its U.S. Food and Drug Administration (FDA) approval in 2024 ref^19,40,41^. This landmark approval validated CLDN18.2 as a therapeutic target and established a foundation for subsequent CLDN-based therapies. A combined analysis of the two Phase III trials showed a median PFS of 9.2 months in the zolbetuximab arm versus 8.2 months in the control arm (chemotherapy alone), and a median OS of 16.4 versus 13.7 months, respectively, with an objective response rate **[G]** (ORR) of 45.5% and a complete response rate **[G]** of 6%. The most common adverse events associated with zolbetuximab were nausea and vomiting, most likely an off-tumor on-target effect related to CLDN18.2 expression in the normal gastric epithelium^19^.

Beyond chemotherapy combinations, zolbetuximab has also been evaluated in combination with immune checkpoint inhibitors in gastroesophageal adenocarcinoma. In the phase II multi-cohort study ILUSTRO, a cohort receiving zolbetuximab with the anti-PD-1 antibody pembrolizumab demonstrated acceptable safety but limited efficacy in a small group of heavily pretreated patients^138^. Larger studies, including the ongoing Phase III LUCERNA trial (NCT06901531 ref^139^) will define the added value of immunotherapy combinations, and early data from agents like the CLDN18.2-targeting antibody TST-001 (osemitamab) have been encouraging^140^. These investigations aim to clarify the potential synergy or cooperativity between CLDN18.2-targeted antibodies and immune checkpoint inhibition, similar to what has been observed in HER2-positive gastroesophageal adenocarcinoma^141^.

Several other therapeutic platforms are also under development. ADCs such as sonesitatug vedotin (formerly AZD0901 or CMG901), IBI343, and SHR-A1904 deliver cytotoxic payloads directly to CLDN18.2-positive cells and have shown promising efficacy in early-phase studies, with ORRs ranging from 24–29%^142–145^. Phase III trials are ongoing in the second-line or later treatment of advanced or metastatic gastric or gastroesophageal junction adenocarcinoma expressing CLDN18.2 (Supplementary Table 1).

Bispecific antibodies and T cell engagers such as Q-1802 ref^146^, ASP2138 ref^147^ and givastomig (bispecific antibodies targeting CLDN18.2 and the co-stimulatory receptor **[G]** 4-1BB, also known as CD137)^148^, act by recruiting T cells through CD3 or modulating immune pathways via PDL1 or 4-1BB to enhance immune-mediated tumor cell killing. In a first-in-human study of givastomig involving patients with metastatic or advanced CLDN18.2-positive solid tumors, an ORR of 16% was observed with manageable toxicity, with hematologic and gastrointestinal toxicities being the most common adverse events^149^. Givastomig is now in clinical development including combination with anti-PD1 or chemotherapy (NCT04900818 ref^150^).

Combination therapies involving immune checkpoint inhibitors and bispecific antibodies and bispecific T-cell engagers have also been explored in preclinical models. Gresonitamab, a half-life–extended bispecific T-cell engager targeting CLDN18.2 and CD3, improved survival in pancreatic cancer xenograft models when combined with anti–PD1 therapy. This combination also resulted in greater tumor infiltration by both activated and exhausted T cells compared to monotherapy with bispecific T-cell engagers ^151,152^.

Finally, CAR T cell therapies have demonstrated clinical activity in CLDN18.2-positive gastroesophageal adenocarcinoma. In a recent open-label Phase II trial involving patients previously treated with CLDN18.2-targeted therapies, CLDN18.2 specific CAR T cells outperformed investigator-choice chemotherapy, achieving an ORR of 35%, a median duration of response of 3.88 months, and a PFS of 3.25 months versus 1.77 months, with OS of 7.92 versus 5.49 months^136^.

These emerging therapeutic modalities collectively broaden the treatment landscape for CLDN18.2-positive tumors and solidify its position as the most mature target in CLDN-directed oncology.

### Leveraging cancer-restricted CLDN6 expression

Similar to CLDN18.2, CLDN6 has limited expression in adult normal tissues, making it a highly tumor-restricted candidate antigen. CLDN6 is selectively expressed in germ cell, endometrial, and ovarian cancers (Fig. 2), which underlies the diverse array of therapeutic approaches being explored. In a phase II trial including patients with relapsed or refractory germ cell tumors, ASP1650, a monoclonal antibody showed limited efficacy as monotherapy^153^ Subsequent clinical trials using ADCs such as DS-9606a (with pyrrolobenzodiazepine payload) and TORL-1-23 (with an MMAE payload) in CLDN6-positive solid tumors have shown more encouraging results as reported in early-phase trials ^154,155^. Bispecific and trispecific formats, including BNT142 and SAIL66, have entered clinical evaluation in CLDN6-positive solid tumors, offering additional functionality such as CD3 engagement and 4-1BB co-stimulation with increased intratumor T cell infiltration^156,157^. Notably, the CAR T cell therapy BNT211 incorporates a CLDN6-specific CAR with a self-amplifying RNA vaccine (CARVac) designed to enhance T cell persistence and antitumor efficacy^158^. Initial results from a phase I/II study in CLDN6-positive solid tumors showed response rates of 33%, albeit with a notable incidence of cytokine release syndrome **[G]** (46%), highlighting both the potential and the challenges of CLDN6-targeted cell therapies^158^.

### Targeting the dual role of CLDN1 in cancer and fibrosis

Unlike CLDN6 and CLDN18.2, CLDN1 is more broadly expressed across tumors such as HCC, cholangiocarcinoma, CRC, non-small cell lung cancer, urothelial cancer, esophageal cancer cervical cancer and HNSCC. Its oncogenic role is driven by surface-exposed, non-junctional expression. The activation of oncogenic intracellular signaling pathways including EMT, MAPK–ERK, and pathways sustaining cancer stemness such as NOTCH1 make CLDN1 an attractive therapeutic target. Therapeutic strategies targeting CLDN1 include mAbs that bind the non-junctional form of the protein exposed on the cancer cell surface, showing potent preclinical activity in such CLDN1-positive cancer models^33^. Notably, CLDN1-targeted therapies have entered early-phase clinical evaluation (phase I/II) for solid CLDN1-positive cancers with CLDN1-directed ADC such as ALE.P02 (MMAE payload, NCT06747585 ref^159^) and ALE.P03 (exatecan payload, NCT07169734 ref^160^) (Supplementary Table 1). Additionally, anti-CLDN1 mAbs exhibit strong antifibrotic effects^16,128^, making them potentially valuable for fibrosis-associated CLDN1-positive cancers such as HCC and intrahepatic cholangiocarcinoma^16^. Notably, CLDN1 targeting is being evaluated outside oncology. The anti-CLDN1 mAb lixudebart (previously known as ALE.F02) is under clinical investigation in advanced liver fibrosis/mild cirrhosis (FEGATO-01; NCT05939947 ref^161^) and in ANCA-associated vasculitis with rapidly progressive glomerulonephritis (RENAL-F02; NCT06047171 ref^162^).

### Targeting CLDN4 in epithelial cancers

CLDN4 is widely expressed in epithelial cancers such as pancreatic, colorectal, ovarian, gastric, bladder, and breast carcinomas. Although not as tumor-restricted as CLDN6 or CLDN18.2, its surface accessibility increases in poorly polarized or dedifferentiated tumors, creating a potential therapeutic window for selective targeting.

The bispecific antibody ASP1002 is the first clinical-stage CLDN4-targeted therapy and is currently being tested in a phase I trial in solid tumors (NCT05719558 ref^163^). By simultaneously engaging CLDN4 and the co-stimulatory receptor 4-1BB, ASP1002 seeks to restrict T cell activation to the TME^164^. Early clinical data will help to understand the feasibility and safety of this conditionally immunostimulatory approach.

In the preclinical setting, multiple anti-CLDN4 mAbs have demonstrated potent ADCC and tumor inhibition in xenograft models of pancreatic, ovarian, gastric and colon cancer^165,166^. The mAb 4D3 enhances paracellular drug penetration and synergizes with low-dose chemotherapy^167^, while the bispecific KM3907 targets both CLDN3 and CLDN4, broadening applicability to dual-expressing tumors^168^.

### CLDN3 and CLDN9 as future therapeutic targets?

CLDN3 is frequently overexpressed in gynecologic and gastrointestinal tumors. ABN501, a first-in-class anti-CLDN3 mAb, has shown ADCC and in vivo efficacy in preclinical models of small-cell lung cancer^169^. Additional strategies include CLDN3 and CD3 bispecific antibodies and bispecific ADCs targeting CLDN3 and epithelial cell adhesion molecule (EpCAM), with deruxtecan topoisomerase I inhibitor payloads demonstrating cytotoxicity in dual CLDN3- and EpCAM expressing tumors in preclinical studies^170,171^.

CLDN9, which is expressed in ovarian, breast, and head and neck cancers, remains at an earlier stage of investigation. As of now, no CLDN9-targeted agents have entered IND-enabling studies or clinical trials.

### Therapeutic strategies acting on CLDN intracellular domains and pathways

While extracellular domains have been the dominant focus of therapeutic development, intracellular CLDN domains, particularly the C-terminal domain and PDZ-binding motifs, represent another avenue for disrupting CLDN-driven oncogenic signaling. These motifs contribute to regulation of tight junction integrity, intracellular signaling and PTMs, and their disruption may complement extracellular-directed approaches.

Small molecules targeting CLDN1, such as N-phenylcarbamothioyl-2-naphthamide derivatives (for example, PDS-0330), have shown preclinical efficacy in CRC models by impairing CLDN1-dependent survival^137,172^. Although their exact binding sites remain undefined, these agents appear to interfere with CLDN1-SRC associations and suggest the potential of intracellular CLDN modulation as a candidate therapeutic strategy. However, these compounds currently remain at an early preclinical stage, and no clinical development or registered clinical trials have been reported to date.

Peptide-based inhibitors have also shown CLDN-targeting mechanistic proof-of-concept. The CLDN1-derived peptide CL58 blocks hepatitis C virus entry in hepatoma cell lines and primary human hepatocytes by competing with native CLDN1 for viral binding^173^. While not yet developed for cancer, this strategy demonstrates how intracellular or transmembrane regions can be mimicked to interrupt protein–protein interactions.

## Knowledge gaps and opportunities in CLDN targeting

While the development of CLDN-targeted therapies marks an important milestone in precision oncology, several challenges remain unresolved. To optimally exploit CLDN targeting deeper mechanistic insights are needed into CLDN function in tumorigenesis. A central question is whether CLDN overexpression acts as a mere epithelial marker or functions as true oncogenic driver. Systematic dissection of CLDN-dependent signaling as well as understanding of the TME interactions are essential to delineate CLDN-driven oncogenic programs and translate these insights into better therapeutic strategies.

Refining biomarker strategies for patient selection will be crucial for successful clinical development. Patient selection represents a critical consideration and CLDN18.2-targeted therapy exemplifies the unmet medical need. The two phase III clinical trials enrolled patients with HER2-negative gastroesophageal cancers exhibiting moderate to strong membranous CLDN18.2 expression in ≥75% of tumor cells. In contrast, the ILUSTRO trial also included patients with intermediate expression (50–75%), but this subgroup demonstrated no objective responses and markedly shorter progression-free survival compared to patients with high expression. Similarly, the phase II FAST trial showed that adding zolbetuximab to chemotherapy significantly improved PFS and OS in patients with CLDN18.2 expression ≥40%^174^. However, subgroup analysis revealed that patients with 40–69% expression did not experience meaningful PFS or OS benefit^174^. These data suggest that inclusion of lower-expression populations may compromise the efficacy of targeted therapies particularly for mAbs.

Nevertheless, with newer modalities such as ADCs, CAR T cells, and bispecific antibodies, there may be potential to treat tumors with lower CLDN18.2 expression. This paradigm parallels the evolution of HER2-targeting in breast cancer, where ADCs extended treatment beyond tumors requiring high HER2 expression by leveraging their cytotoxic payload delivery and bystander effects, targeting adjacent tumor cells even when target expression is low or heterogeneous^175–177^. Bispecific antibodies may also be effective at lower antigen thresholds, as they recruit and activate T cells upon minimal antigen engagement, enabling cytotoxic activity without requiring high antigen density or target internalization^178^. This is now being studied clinically, for example the CLDN18.2-targeted CAR T cell trial included patients with moderate staining in at least 40% of tumor cells, although the majority had high expression levels^136^. This approach could broaden the treatable population, increasing the percentage of eligible patients with gastric cancer from approximately 40%^40,41^ to nearly 60%^131^. Similarly, Phase I ADC trials have enrolled patients with as little as 1% CLDN18.2-positive cells^142,143^. While lowering the positivity threshold introduces challenges: intratumoral heterogeneity needs to be captured, treatment efficacy could decline and the risk of resistance or CLDN-negative clone selection may increase^145^. To better understand resistance mechanisms, clinical data and post-treatment biopsies are essential. A recent clinical study suggests that CLDN18.2 expression is largely retained after treatment with the CLDN18.2 antibody zolbetuximab, suggesting that sequential targeting strategies may be viable^179^.

The methodology used to evaluate CLDN expression also affects patient selection. Although most current clinical trials rely on conventional IHC to define CLDN expression, this method can lead to subjective and inconsistent results across studies^180^. The H-score system offers semi-quantitative information incorporating both staining intensity and extent, and can be automated, but its use is inconsistent, and thresholds are not always standardized^181–183^. Additionally, tissues microarrays (TMAs), although practical for high-throughput screening, may fail to accurately capture intratumoral spatial heterogeneity in CLDN expression and localization^58^. Spatial profiling may help to shed light on the biological processes operating in CLDN-positive and CLDN-negative tumor regions. Combined with quantitative approaches for CLDN detection, it can help to develop a biomarker companion for clinical development.

Although the relatively low normal–to–tumor expression ratio may appear to limit concerns, the management of on-target, off-tumor toxicity remains a critical translational issue. CLDNs are not exclusively expressed by tumor cells increasing the risk of unintended toxicities when targeted therapeutically. Consequently, data from clinical trials are essential to evaluate safety profiles and to characterize potential on-target, off-tumor toxicities. To date, most clinical data pertain to CLDN18.2-targeting therapies. Zolbetuximab, has demonstrated on-target specific toxicity, primarily gastrointestinal toxicities, while ADCs and CAR T cell therapies are associated predominantly with hematologic toxicities related to the therapeutic modality itself (for example cytokine release syndrome with CAR T cell therapy, or payload-specific toxicities with ADCs) alongside an increase in gastrointestinal events^40,41,136,138,142,143^. CLDN6, which exhibits low expression in healthy adult tissues, has been evaluated in early-phase trials involving both mAbs and CAR T cells. These studies have primarily reported hematologic toxicities related to lymphodepletion, as well as asymptomatic elevations in transaminases and lipase levels^153,158^. In comparison, CLDN1 is highly expressed in the skin, liver (Table 1), and in kidneys is expressed in Bowman’s capsule^184^, and CLDN4 is predominantly found in the gastrointestinal tract (Table 1). This expression pattern suggests potential concerns for dermatologic, gastrointestinal, renal adverse effects. However, preclinical studies involving anti-CLDN1 antibodies and CLDN4-targeting therapies have not identified any major toxicity signals to date. For CLDN1, the mAb lixudebart (ALE.F02) has been evaluated in a repeat-dose regimen consisting of 4 weekly IV infusions in non-human primate with no major safety signals reported.^16^ For CLDN4, safety data are derived from treatment studies using mouse xenografts models^167,168^. Taken together, these data emphasize that the balance between therapeutic efficacy and off-tumor toxicity is highly dependent on the physiological distribution of the targeted claudin but also on the therapeutic format used.

The therapeutic landscape for CLDN-targeted interventions is evolving, offering promising avenues for clinical application. Combination strategies represent a major area of expansion. Clinical trials with Zolbetuximab have shownthat combining CLDN18.2-targeted therapy with chemotherapy is more effective than using the antibody alone^40,41,138,185^. Early-phase clinical data on combination therapies involving immunotherapy, with or without chemotherapy, alongside ADCs and bispecific antibodies targeting CLDN18.2 suggest that CLDN-directed therapies may sensitize tumors to chemotherapy, and that these combinations are safe^140,186^. Rational combinations with immune checkpoint inhibitors, TME modulators, or epigenetic drugs could address immune exclusion and/or heterogeneity. CLDN1-targeting agents, in particular, have shown antifibrotic properties and the capacity to remodel the TME in HCC and cholangiocarcinoma models^16,33^. These effects may extend beyond tumor cytotoxicity to disease modification by reversing stromal remodeling, restoring immune infiltration, and improving response to checkpoint blockade. These hypotheses require further study to explore their translational relevance and to determine whether they support broader indications, including fibrosis-driven pre-malignant states.

## Conclusions

CLDN-targeted therapies reflect the translation of decade-long preclinical investigation converging the knowledge of tumor biology and biomarker-driven precision medicine. Zolbetuximab’s approval for CLDN18.2-positive gastroesophageal cancer underscores the clinical viability of this class, while active programs targeting CLDN1, CLDN3, CLDN4 and CLDN6 are ongoing. The diversity of platforms (mAbs, ADCs, bispecifics, CAR T cells) and the integration of intracellular-targeting strategies provide multiple avenues for innovation. As the field advances, key challenges will include defining CLDN expression thresholds for patient selection, minimizing off-tumor toxicity, and exploring rational combinations with checkpoint inhibitors or RNA-based vaccines. With continued preclinical progress and clinical expansion, CLDN-targeted therapies are poised to become foundational components in the treatment landscape for solid tumors, particularly in cancers with distinct CLDN expression profiles.

## Supplementary Material

Supplementary information is available for this paper at https://doi.org/10.1038/s415XX-XXX-XXXX-X

## Figures and Tables

**Table 1:** Overview of human CLDN family members and their main biological functions.

Human Claudin (CLDN)^a)^	Main Expression Sites ^b)^	Primary Biological Function	Other Functions and Related Diseases
**CLDN1**	Liver, skin	Autonomous barrier-forming tight-junction^12^.	Receptor for hepatitis virus C^187^. Mutation is associated with neonatal ichthyosis-sclerosing cholangitis (NISCH) syndrome^188^
**CLDN2**	Choroid plexus, kidney, gallbladder	Highly cation-selective and water-permeable paracellular channels^12,189^.	Mutation causes obstructive azoospermia with nephrolithiasis (OAZON) syndrome^190^
**CLDN3**	Colon, small intestine	Autonomous barrier-forming tight-junction^12^.	Receptor for Clostridium perfringens enterotoxin^191^
**CLDN4**	Esophagus, urinary bladder, colon, salivary gland, skin	Autonomous barrier-forming tight-junction^12^. Interacts with CLDN8 to form chloride channels in the kidney^192^.	Receptor for Clostridium perfringens enterotoxin^193^
**CLDN5**	Adipose tissue, breast, choroid plexus, lung	Autonomous barrier-forming tight-junction^12^.	Major component of the blood–brain barrier^194^. Pathogenic sequence variants cause a neurodevelopmental disorder characterized by seizures, microcephaly and brain calcifications^195^.
**CLDN6**	Basal ganglia, pancreas, testis, cerebellum, placenta	Autonomous barrier-forming tight-junction^12^. Lowly expressed.	Oncofetal CLDN; involved in epithelial differentiation^196^.
**CLDN7**	Esophagus, colon, small intestine	Autonomous barrier-forming tight-junction^12,197^.	NA
**CLDN8**	Kidney, breast, rectum, salivary gland	Non-autonomous barrier-forming tight-junction **[G]**^12,197^.	NA
**CLDN9**	Pituitary gland, cerebellum	Autonomous barrier-forming tight-junction^12^	Required for cochlear ion homeostasis; mutations cause nonsyndromic deafness^198^.
**CLDN10**	Pancreas, salivary gland, kidney	Isoform-specific ion-selective paracellular channels^199^. CLDN10a forms anion-selective channels while CLDN10b forms low cation-selective channels^12^.	Defective mutation in CLDN10B cause Xelix syndrome^200^.
**CLDN11**	Spinal cord	Autonomous barrier-forming tight-junction^12^	Maintains electrical insulation in myelin; essential for Sertoli cell tight junctions and fertility^201^. Stop-loss variants in CLDN11 cause hypomyelinating leukodystrophy^202^
**CLDN12**	Liver, retina	Non-autonomous barrier-forming tight-junction^12^ lacking the PDZ-binding motif^203^. Putative role in calcium permeability^204^.	NA
**CLDN14**	Liver	Autonomous barrier-forming tight-junction with a low anion-selective paracellular channel activity^12^.	Mutations cause non syndromic recessive deafness DFNB29^205^.
**CLDN15**	Duodenum, small intestine	Moderately cation-selective and water-permeable paracellular channel^12,206^.	NA
**CLDN16**	Kidney	Non-autonomous barrier-forming tight-junction^12^. Forms heteromeric channels with CLDN19 and regulates Mg^2^^+^ and Ca^2+^ reabsorption^207^	Mutations cause familial primary hypomagnesemia with hypercalciuria and nephrocalcinosis (FHHNC)^208^.
**CLDN17**	Vagina, cervix, esophagus, salivary gland	Non-autonomous barrier-forming tight-junction^12^ with anion-selective paracellular channel activity^209^.	NA
**CLDN18**	Stomach	Autonomous barrier-forming tight-junction which exists in two isoforms: CLDN18.1 (lung-specific)^210^ and CLDN18.2 (stomach-specific)^210,211^ which its barrier strength increases under acidic condition^12^.	NA
**CLDN19**	Kidney	Autonomous barrier-forming tight-junction with a low cation-selective paracellular channel activity^12^. Forms heteromeric channels with CLDN16.	Mutations cause FHHNC and severe ocular impairment^212^.
**CLDN20**	Retina, bone marrow, skin, spinal cord, smooth muscle	Non-autonomous barrier-forming tight-junction^12^. Lowly expressed.	NA
**CLDN22**	Salivary gland	Non-autonomous barrier-forming tight-junction^12^.	NA
**CLDN23**	Colon, stomach, skin, liver, small intestine	Non-autonomous barrier-forming tight-junction^12^. Forms heteromeric and heterotypic complexes with CLDN3 and CLDN4 and 3 regulates paracellular ion and macromolecule permeability^213^.	NA
**CLDN24**	Kidney	Non-autonomous barrier-forming tight-junction^12^. Lowly expressed.	NA
**CLDN25**	Pituitary gland	Non-autonomous barrier-forming tight-junction^12^. Lowly expressed.	Mutations have been associated with Pelizaeus-Merzbacher-like leukodystrophy **[G]^214^**
**CLDN34**	Testis	Unknown	NA

NA, not applicable

a) CLDN13 is absent in humans. CLDN26 and CLDN27 are predicted genes with low or no confirmed expression in human tissues; therefore, they are not included here and are also not recognized as a part of the CLDN family by the HUGO Gene Nomenclature Committee at the University of Cambridge. CLDN34 has been validated at the protein level; however, it is not classified within the CLDN family by the HUGO Gene Nomenclature Committee.

b) Main tissue expression sites were derived from the Human Protein Atlas (v25.0) consensus RNA dataset. For each CLDN gene, the tissue maximum nTPM (normalized Transcripts Per Million) was identified and up to five tissues were retained if their nTPM values were within 50% of this maximum (≥0.50×max). Low expression refers to CLDNs with a maximum nTPM value of 1 or less.

**Table 2:** Overview of human CLDN family members expression and function in cancer.

Claudin (CLDN)	Detection across cancer types ^a)^	Cancers with specific enrichment or enhancement ^b)^	Predominant cancer role	Mechanistic data ^c)^	Confidence^d)^	Examples for cancer types with functional role	ref
CLDN1	Detected in all	None	Tumor promoting	Yes	High	CRC, HCC, CCA	32,33,109
CLDN2	Detected in many	Papillary RCC	Tumor promoting	Yes	Moderate	CRC	27,104,215
CLDN3	Detected in many	Serous cystadenocarcinoma	Tumor promoting	Limited	Low	Ovarian cancer	118,127,216,217
CLDN4	Detected in many	None	Lineage marker	Limited	Low	Pancreatic cancer, Ovarian cancer	81,127,218,219
CLDN5	Detected in all	GBM	Vascular barrier permeability	Limited	Low	GBM, HCC, Lung metastasis	220–223
CLDN6	Detected in many	Serous Cystadenocarcinoma; Testicular Germ Cell Tumor; Endometrial cancer	Tumor promoting,	Yes	High	Endometrial cancer, Gastric cancer, HCC	17,83,91–93
			Tumor suppressing		Low-moderate	Breast cancer	92,224,225
CLDN7	Detected in all	None	Tumor promoting	Limited	Low-moderate	CRC and pancreatic cancer.	96,226
			Tumor suppressing		Moderate	CRC, RCC, breast cancer, lung cancer	94,95,227–230
CLDN8	Detected in many	Chromophobe RCC; Prostate Adenocarcinoma	Tumor promoting	Limited	Low	RCC, breast cancer, CRC	105,231,232
CLDN9	Detected in many	Serous Cystadenocarcinoma	Tumor promoting	Limited	Low	Gastric cancer, HCC	233,234
CLDN10	Detected in many	Pancreatic Adenocarcinoma	Tumor promoting	Limited	Low	RCC, Thyroid cancer	235–237
			Tumor suppressing		Moderate	RCC	236
CLDN11	Detected in some	GBM	Tumor promoting,	Limited	Moderate	SCC, Lung cancer	115,116
			Tumor suppressing		Low	Gastric cancer, Bladder cancer	238,239
CLDN12	Detected in all	None	Prognosis; context-depedent	Limited	Low	Lung cancer, breast cancer	240–243
CLDN14	Detected in many	HCC; Cutaneous Melanoma	Tumor promoting	Limited	Low	Lung cancer, CRC	244–246
CLDN15	Detected in all	None	Insufficient functional evidence	-	-	-	-
CLDN16	Detected in many	Ovary Serous Cystadenocarcinoma; Thyroid Carcinoma	Insufficient functional evidence	No	Low	-	-
CLDN17	Detected in single	HNSCC	Tumor suppressing	Limited	Low	HNSCC	247
CLDN18	Detected in some	Pancreatic Adenocarcinoma; Stomach Adenocarcinoma	CLDN18.1: Tumor suppressor	Limited	Low	Lung cancer	106
			CLDN18.2: Lineage marker; tumor promoting	Yes	Moderate		248,249
			CLDN18-ARHGAP26 (fusion): Tumor promoting	Yes	High	Diffuse gastric cancer	69,250
CLDN19	Detected in many	Testicular Germ Cell Tumor	Tumor suppressor	Limited	Low	Breast cancer	251
CLDN20	Detected in single	Lung SCC	Tumor promoting	Limited	Low	Breast cancer	252
CLDN22	Detected in some	Serous Cystadenocarcinoma; Endometrial cancer	Insufficient functional evidence	-	-	-	-
CLDN23	Detected in all	None	Tumor suppressor	No	Low	Gastric cancer	253
CLDN24	Not detected	Not detected	Insufficient functional evidence	-	-	-	-
CLDN25	Not detected	Not detected	Insufficient functional evidence	-	-	-	-

a Data from the Human Protein Atlas (HPA) v24 analysis of TCGA RNA data. Gene detection is categorized based on the number of tissues, regions, or cell types in which expression is observed. Detected in single: present in only one type one cancer type. Detected in some: more than one but less than one-third of cancers. Detected in many: at least one-third but not all cancers. Detected in all: all cancer types. Not detected: normalized transcripts per million (TPM) < 1 across all cancer types.

b Data from HPA v24 analysis of TCGA RNA data. Each cancer sample was rescaled so that the total TPM across all genes equaled 1,000,000 (TPM for protein-coding genes, pTPM). Enrichment was defined as pTPM in a particular cancer type being at least four times higher than in any other cancer type. Enhancement as pTPM in one or several cancer types that has at least four times the average expression of all cancer types.

c Mechanistic data denotes experimental proof that a CLDN causally drives or suppresses cancer phenotypes, shown by targeted loss- or gain-of-function with appropriate controls (e.g., rescue or epistasis), pathway readouts, or in vivo validation. Grading: Yes when causal perturbation with rescue or epistasis is reproduced across models or in vivo; Limited when causality is suggested but confined to in vitro or single-model or partial pathway evidence; No when data are correlative only without targeted perturbation.

d Confidence reflects the weight and reproducibility of evidence, prioritizing mechanistic data (gain or loss-of-function and rescue experiments), validation across relevant in vivo model systems, consistency across independent studies; high = patient-derived models, coherent mechanistic data and in vivo validation, moderate = in vitro patient-derived models without in vivo validation or models limited to cell lines and cell line-derived xenografts, low = models limited to cell lines or correlative data. ARHGAP26, Rho GTPase–activating protein 26; CCA, cholangiocarcinoma; CRC, colorectal cancer; GBM, glioblastoma multiforme; HCC, hepatocellular carcinoma; HNSCC, head and neck squamous cell carcinoma; HPA, Human Protein Atlas; RCC, renal cell carcinoma; TCGA, The Cancer Genome Atlas; TPM, transcripts per million.

## Glossary

Non-autonomous barrier-forming tight junction: a tight junction that does not independently create a paracellular barrier but instead requires integration into preexisting tight junctions formed by other claudins to become functional.
Tight junction strand: A fibrillar sealing structure at the apical cell–cell contact, formed mainly by polymerized claudins and associated tight junction proteins, that underlies the paracellular diffusion barrier.
Signet-ring cell carcinoma: A poorly cohesive, mucin-rich adenocarcinoma in which intracytoplasmic mucin displaces the nucleus, producing “signet-ring” cells.
Cyclic hypoxia: Recurrent, transient fluctuations in tumor oxygenation over time, driven by unstable perfusion, that promote adaptive stress responses.
Epithelial–mesenchymal transition: A reversible cellular program in which epithelial cells lose polarity and cell–cell adhesion and acquire mesenchymal traits that enhance motility and invasiveness.
Claudin-low breast cancer: A molecular subtype of breast cancer characterized by low expression of tight junction and associated with mesenchymal and stemness features.
Plasticity: The capacity of cancer cells to reversibly switch phenotypic states in response to intrinsic signals or microenvironmental pressure.
Biliary fibrosis: Pathological accumulation of extracellular matrix around bile ducts, typically driven by chronic cholangiocyte injury and inflammation, leading to progressive ductal damage and liver fibrosis.
Immune checkpoint inhibitors: Therapeutic antibodies that block inhibitory immune pathways (for example, PD-1/PD-L1 or CTLA-4) to restore anti-tumor T cell activity.
Combined positive score: An immunohistochemistry metric for PD-L1 that quantifies the proportion of PD-L1–positive cells, including immune cells, relative to total viable tumor cells.
Antibody-dependent cell-mediated cytotoxicity: Fc receptor–dependent killing of antibody-opsonized target cells by immune effector cells, most notably NK cells.
Complement-dependent cytotoxicity: Antibody-triggered activation of the complement cascade that forms membrane attack complexes and lyses target cells.
Co-stimulatory receptor: An activating receptor on immune cells (commonly T cells) that provides a “second signal” required for full activation, expansion, and effector function.
Cytokine release syndrome: An acute systemic inflammatory toxicity caused by rapid cytokine release after immune activation, ranging from fever to life-threatening shock.
Pelizaeus–Merzbacher-like leukodystrophy: A rare autosomal recessive hypomyelinating leukodystrophy resembling Pelizaeus–Merzbacher disease, characterized by early-onset neurological impairment due to defective central nervous system myelin formation
Anoikis: A form of programmed cell death triggered when epithelial cells detach from the extracellular matrix.
